# Editorial: Updates in ocular therapeutics and surgery, volume III

**DOI:** 10.3389/fmed.2024.1536632

**Published:** 2025-01-13

**Authors:** Georgios D. Panos, Horace Massa

**Affiliations:** ^1^First Department of Ophthalmology, AHEPA University Hospital, School of Medicine, Aristotle University of Thessaloniki, Thessaloniki, Greece; ^2^Division of Ophthalmology and Visual Sciences, School of Medicine, University of Nottingham, Nottingham, United Kingdom; ^3^Department of Ophthalmology, Geneva University Hospitals, Geneva, Switzerland

**Keywords:** cornea, macular oedema, uveitis, glaucoma, vitreoretinal surgery, machine learning, eyelid surgery, paediatric ophthalmology

Ophthalmology stands as one of the most rapidly evolving fields in medicine, characterised by groundbreaking advancements in therapeutics and surgery over the last few decades. The introduction of transformative treatments for ocular conditions, such as age-related macular degeneration, glaucoma, retinal vein occlusions, diabetic macular oedema, genetic disorders, and uveitis, has significantly enhanced patient outcomes and reshaped clinical paradigms. Simultaneously, technological innovations and refined surgical techniques have ushered in a new era for eye surgeons, enabling procedures that are safer, faster, and more precise than ever before.

This Research Topic, *Updates in ocular therapeutics and surgery, volume III*, builds upon the foundation of its successful predecessors, continuing the journey of exploring the latest therapeutic and surgical advances. It aims to consolidate contemporary knowledge, highlight cutting-edge research, and chart the trajectory of future developments in the field.

The contributions in this Research Topic underscore the dynamic interplay between clinical innovation and translational research. They reflect the commitment of ophthalmologists, researchers, and industry pioneers to address complex ocular diseases with novel solutions while enhancing the efficacy and safety of surgical interventions.

By bringing together a diverse array of studies and reviews, this Research Topic aspires to serve as a valuable resource for clinicians, researchers, and trainees. It not only encapsulates the current state-of-the-art in ocular therapeutics and surgery but also seeks to inspire future inquiries that will further refine the practice of ophthalmology.

Guo et al. investigated the therapeutic potential of Urolithin A in addressing delayed corneal epithelial wound healing. By inhibiting ferroptosis, a form of oxidative stress-induced cell death, Urolithin A demonstrated significant promise in mitigating hyperosmotic stress and promoting healing.

Li et al. evaluated the efficacy and safety of CO_2_ laser-assisted sclerectomy for secondary glaucoma following vitrectomy. Their findings underscored the advantages of this novel approach in achieving substantial intraocular pressure reduction with a lower risk of complications. Similarly, He et al. performed a systematic review and meta-analysis exploring the synergistic effect of anti-VEGF therapy combined with Ahmed valve implantation in neovascular glaucoma, revealing enhanced surgical success rates and improved long-term outcomes compared to valve implantation alone.

Liu et al. presented an innovative approach to managing Descemet's membrane detachment post-cataract surgery. Their technique of using air tamponade offered a minimally invasive and highly effective solution to this challenging complication. Ning et al. contributed a bibliometric analysis of 20 years of research on posterior chamber phakic intraocular lenses (pIOLs), identifying key research areas including clinical outcomes, complications, and postoperative visual quality. This comprehensive analysis provides valuable insights for advancing the field.

Wang and Zheng introduced the Prism and Maddox Rod Test as a reliable and cost-effective tool for determining surgical measurements in type III acute acquired comitant esotropia. Their work demonstrated its precision and applicability in improving alignment outcomes post-surgery.

In a review of corneal epithelial repair mechanisms, Gong et al. detailed the critical roles of growth factors such as epidermal growth factor and transforming growth factor-α, highlighting their therapeutic potential in ocular surface disease management.

Ye et al. proposed a modified technique for the intrascleral fixation of three-piece foldable intraocular lenses using Hoffman pockets. This approach minimised complications and improved lens stability, offering a refined method for secondary IOL implantation.

In the domain of ocular trauma, Sather et al. examined the outcomes of secondary surgeries following open globe injury repair. Their findings emphasised the importance of a structured surgical rehabilitative process, with ~50% of patients achieving ambulatory vision and 30% attaining reading vision.

Körber et al. explored the use of picosecond ultrashort laser pulses in ophthalmic surgeries, showcasing their superior precision and reduced collateral damage compared to traditional nanosecond lasers. Their research paves the way for safer and more efficient laser-based procedures.

Finally, Jin et al. investigated the efficacy and safety of platelet-rich plasma (PRP) in acute non-arteritic anterior ischaemic optic neuropathy (NAION). PRP treatment significantly improved capillary perfusion of the optic nerve head and short-term visual acuity, demonstrating its potential as a novel therapeutic option for this challenging condition.

This Research Topic of studies reflects the vibrancy and innovation in ocular therapeutics and surgery. We hope that readers will find this volume as engaging and enlightening as we did in editing and presenting it, and that it inspires future endeavours aimed at further refining the care of patients with ocular diseases.

